# Reading Comprehension Predictors in European Portuguese Adults

**DOI:** 10.3389/fpsyg.2021.789413

**Published:** 2021-12-02

**Authors:** Fábio Gonçalves, Alexandra Reis, Filomena Inácio, Inês Salomé Morais, Luís Faísca

**Affiliations:** ^1^Faculdade de Ciências Humanas e Sociais, Departamento de Psicologia e Ciências da Educação, Universidade do Algarve, Faro, Portugal; ^2^Center for Research in Health Technologies and Information Systems (CINTESIS-UAlg), Universidade do Algarve, Faro, Portugal

**Keywords:** reading comprehension, simple view of reading, path-analysis, adult typical readers, European Portuguese

## Abstract

Research on the predictors of reading comprehension has been largely focused on school-aged children and mainly in opaque orthographies, hindering the generalization of the results to adult populations and more transparent orthographies. In the present study, we aim to test two versions of the Simple View of Reading (SVR): the original model and an extended version, including reading fluency and vocabulary. Additional mediation models were analyzed to verify if other reading comprehension predictors (rapid automatized naming, phonological decoding, phonological awareness, morphological awareness, and working memory) have direct effects or if they are mediated through word reading and reading fluency. A sample of 67 typical adult Portuguese readers participated in this study. The SVR model accounted for 27% of the variance in reading comprehension, with oral language comprehension displaying a larger contribution than word reading. In the extended SVR model, reading fluency and vocabulary provided an additional and significant contribution of 7% to the explained variance. Moreover, vocabulary influenced reading comprehension directly and indirectly, *via* oral language comprehension. In the final mediation model, the total mediation hypothesis was rejected, and only morphological awareness showed a direct effect on reading comprehension. These results provide preliminary evidence that the SVR (with the possible addition of vocabulary) might be a reliable model to explain reading comprehension in adult typical readers in a semitransparent orthography. Furthermore, oral language comprehension and vocabulary were the best predictors in the study, suggesting that remediation programs addressing reading comprehension in adults should promote these abilities.

## Introduction

Reading comprehension is the ultimate goal of reading, although it remains an understudied subject when compared to word-level processes (Barquero and Cutting, 2021). One can define reading comprehension as the ability to draw and construct meaning from the text (Snow and RAND Reading Study Group, 2002) through an interactive process whereupon the reader extracts explicit information or infers implicit information through textual cues or the activation of background knowledge (Day and Park, 2005). Adequate reading comprehension is essential for academic achievement, social and cultural participation, and successful functioning in contemporary societies (Cavalli et al., 2019; Hjetland et al., 2020).

Despite its central importance in adults’ everyday life, most reading comprehension studies focus on children, both with and without learning disorders (Earle and Del Tufo, 2021). However, children and adults might differ significantly in the way they achieve reading comprehension. Adults have been exposed to a larger quantity of textual material, because of their extended life experience. Adults also have a greater understanding of the different domains, such as vocabulary, morphological and syntactic knowledge, and logical reasoning, that support comprehension (Thompkins and Binder, 2003). On the other hand, children allocate most of their cognitive resources to decoding, since they are still learning the rules of grapheme-phoneme conversion, leaving fewer resources available for meaning extraction. The allocation of cognitive resources to comprehend seems therefore to be different in these age groups. Greenberg et al. (2002) compared adult literacy students to school-aged children, matched for reading level. When analyzing the groups’ performance on word and non-word reading, spelling, and rhyme word detection tasks, the authors found that children relied mostly on phonological skills, whereas adults were more likely to call upon orthographic knowledge and visual memory strategies. Thus, when confronted with a word that could not be immediately read, children would try to read it through grapheme-phoneme conversion, while adults would typically try to guess the word by comparing it to other words stored in their lexicon. The use of distinct strategies by adults and children might reflect the different cognitive processes that children and adults rely on when reading. Models of reading comprehension should therefore take these differences into account since models developed for children might not be appropriate for adults.

The Simple View of Reading (SVR; Gough and Tunmer, 1986) is a prominent model of reading comprehension, based on English-speaking school-aged children, that has been applied to adults. The SVR postulates that decoding accuracy and oral language comprehension can account for all the variance in reading comprehension: while decoding skills translate print into oral language, oral language comprehension skills make sense of what is read (Gough and Tunmer, 1986). In children, this combination has been shown to capture between 65 and 85% of the variance in reading comprehension (Catts et al., 2005). In adults, the SVR model accounted for a somehow smaller fraction of the reading comprehension variance (34% for a sample of college students; Macaruso and Shankweiler, 2010; and between 64 and 74%, for samples of struggling adult readers; Braze et al., 2007; Sabatini et al., 2010; Talwar et al., 2020).

However, the SVR has often been considered too “simple” to explain such a complex construct as reading comprehension and, consequently, several authors have proposed augmented versions of the original model. Catts (2018) identified two main research lines that argue for expanding the SVR model by adding vocabulary and reading fluency, respectively. Vocabulary is a subcomponent of oral language comprehension, and there is no consensus if its contribution should be subsumed within oral language comprehension or be considered as a distinct component on its own. Gottardo et al. (2018) “unpacked” oral language comprehension into three subcomponents (vocabulary, morphology, and syntax) and found that each of them captured both unique and shared amounts of variance in reading comprehension. Using hierarchical regression models, Braze et al. (2007) also found that vocabulary accounted for unique variance in young adults reading comprehension, independently from word reading and oral language comprehension, thus supporting the addition of vocabulary to the SVR. However, more recent studies, using latent variable analyses, found that the effect of vocabulary on reading comprehension was completely captured by oral language comprehension (Braze et al., 2016; Talwar et al., 2020), thus supporting the opposite view that vocabulary should not be added to the SVR model as a separate component, at least in adults. These contradictory results fail to clarify the role of vocabulary in the SVR, in adults, leaving the issue unresolved.

The SVR model has also been criticized for only considering decoding accuracy but not a speed component such as reading fluency (Fernandes et al., 2017). In children, the inclusion of reading fluency in the SVR yielded inconsistent results, depending on school grade or orthographic transparency (Catts, 2018). In struggling adult readers, both Braze et al. (2007) and Sabatini et al. (2010) found that reading fluency did not provide an additional and significant contribution to reading comprehension, beyond word reading and oral language comprehension. Mellard et al. (2010) used a path analysis approach to test an extended version of the SVR model in low literacy adults and showed that while word reading accuracy had the strongest direct influence on reading comprehension, reading fluency made the second strongest direct contribution, being greater than the oral language comprehension own contribution. Additionally, Macaruso and Shankweiler (2010) found, in a college students’ sample, that reading fluency was the only predictor that accounted for unique variance in reading comprehension over and above decoding and listening comprehension. It seems that, for both children and adults, the role of reading fluency in the SVR is controversial.

The SVR also postulates that, as the reader acquires expertise, it is expected that the main source of variability in reading comprehension shifts from decoding accuracy to oral language comprehension skills (Hoover and Gough, 1990). This shift might be explained by the Perfetti’s Verbal Efficiency Theory (Perfetti, 1985). According to this theory, the cognitive system has limited capacity for decoding and comprehension simultaneously; only when the reader can decode accurately and fluently, the cognitive system can allocate sufficient free attentional resources to the extraction of meaning from the text. Indeed, Catts et al. (2005) found that the contribution of oral language comprehension to reading comprehension increases, while decoding accuracy contribution decreases, as the child progresses through schooling and acquires reading experience. During adolescence, word reading no longer appears to be an important predictor of individual differences in reading comprehension (Foorman et al., 2015).

Nevertheless, and according to Florit and Cain (2011), this shift from decoding accuracy to oral language comprehension seems to be affected by the transparency of the orthographic system. In more opaque orthographies, learning grapheme-phoneme conversion rules is an arduous process, making fluent reading possible only in later school years (Seymour et al., 2003). Subsequently, decoding accuracy stays as the main source of variability in reading comprehension until later in school, when it begins to be replaced by oral language comprehension (Catts et al., 2005). On the other hand, in more transparent orthographies, grapheme-phoneme conversion is simpler, allowing readers to achieve fluent decoding earlier, and therefore being able to focus on comprehension. In a study addressing reading comprehension in European Portuguese (a semitransparent orthography), results showed that for children in the second and fourth grades, oral language comprehension was the strongest contributor to reading comprehension when compared to decoding (Cadime et al., 2017). Also, in transparent orthographies such as Finnish (Torppa et al., 2016) and Italian (Tobia and Bonifacci, 2015), oral language comprehension comes up as the main source of variability in reading comprehension already in early grades, maintaining its preponderant influence as the individual progresses through schooling. These studies add evidence to the suggestion of Florit and Cain (2011) that the transparency of orthography favors the early contribution of oral language comprehension to reading comprehension.

Besides the two components of the SVR (word reading and oral language comprehension), plus the two usual additions to this model (vocabulary and reading fluency), several other predictors have been considered as relevant for adult reading comprehension. Given the lack of consensus about the relative importance of such reading comprehension predictors, Tighe and Schatschneider (2016) performed a meta-analysis of the available literature and identified 10 constructs that should be considered: morphological awareness, language comprehension, reading fluency, oral vocabulary knowledge, real word decoding, working memory, pseudoword decoding, orthographic knowledge, phonological awareness, and rapid automatized naming (RAN). Although only using correlational evidence from a small number of studies, this is the first systematic review addressing the most important reading-related predictors of reading comprehension in adulthood, and it reveals the importance of considering other predictors to reading comprehension beyond the ones assumed by the SVR model (both standard and typically extended versions).

**Figure 1:**
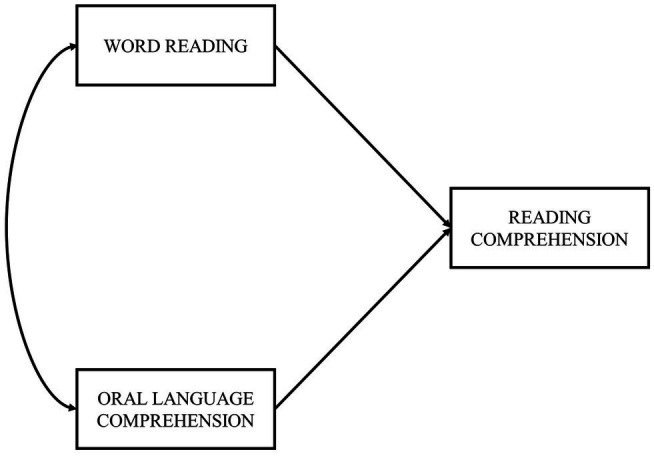
SVR model.

**Figure 2:**
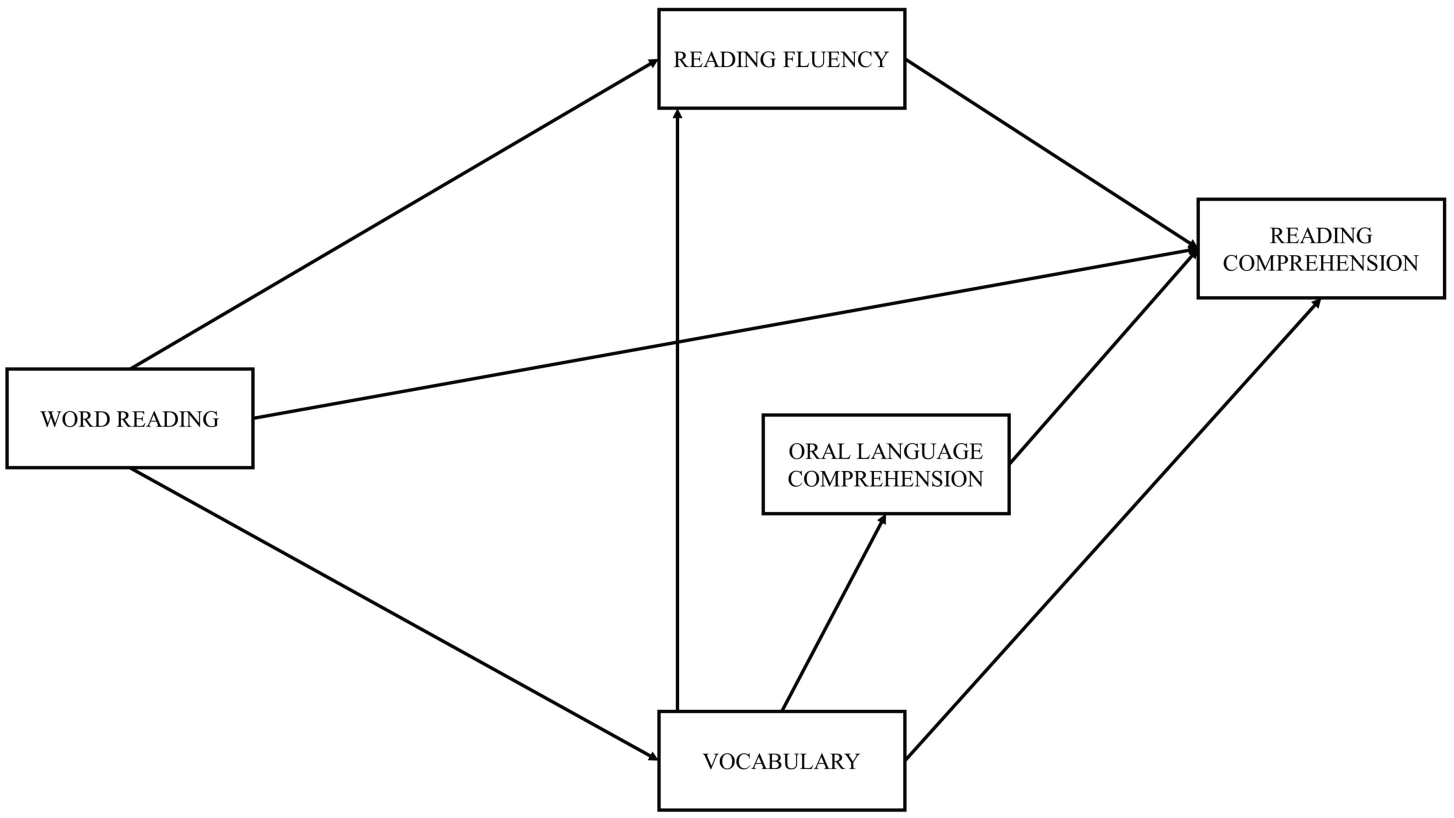
Extended SVR model.

In the present study, we aim to examine the relevance of several predictors to reading comprehension in European Portuguese adult typical readers. Therefore, the SVR model (Figure 1) and an extended SVR model (Figure 2) were tested, the latter including the addition of vocabulary and reading fluency components. Following the suggestion of Tighe and Schatschneider (2016) regarding the most relevant predictors, we also included measures of RAN, phonological decoding, phonological awareness, morphological awareness, and working memory. Measures of orthographic knowledge were not included in the analysis due to excessive low reliability. These variables were tested for their direct and indirect effects on reading comprehension (through both reading measures: word reading and reading fluency).

For the SVR model (Figure 1), we expect that oral language comprehension contributes more to reading comprehension than word reading. Since European Portuguese is a relatively transparent orthography for reading, fluent decoding is expected in adult typical readers and, consequently, the main source of variability in reading comprehension would probably be oral language comprehension individual differences.

In the extended SVR model (Figure 2), word reading appears as an exogenous variable, with paths leading to reading fluency, vocabulary, and reading comprehension. The more accurate the reader is, the faster he is expected to read (Fernandes et al., 2017), thus explaining the first predicted path. Word reading experience contributes to the acquisition of new word meanings, both in context and isolated (Duff et al., 2015), and thus we predict a path from word reading to vocabulary. Lastly, the path from word reading to reading comprehension expresses the role of decoding accuracy in the SVR (Gough and Tunmer, 1986). Reading fluency was considered as an intermediate variable, with a path leading to reading comprehension because when reading is fluent, the cognitive system can free enough attentional resources for the reader to focus on comprehension (Perfetti, 1985; Fernandes et al., 2017). Vocabulary was another intermediate variable in the model, with paths leading to reading fluency, oral language comprehension, and reading comprehension. A larger lexicon leads to a greater number of words the reader understands and recognizes, contributing to a more fluent reading (Kirby et al., 2008; Yildirim et al., 2013). Moreover, vocabulary is known to influence comprehension, since the knowledge of a word’s meaning in context aids in understanding and inference making, both in oral and written modalities (Braze et al., 2007). Oral language comprehension was the last intermediate variable considered. Only one path was tested, from oral language comprehension to reading comprehension, reflecting the role of oral language comprehension in the original SVR model.

The remaining predictors were tested for their putative mediated effects on reading comprehension. Thus, we hypothesized that the effects of RAN, phonological decoding, phonological awareness, and working memory on reading comprehension are completely mediated by word reading and reading fluency. This prediction arises from the role of such variables in word reading as well as the absence of evidence for their direct effects on reading comprehension in typical adult readers. Conversely, there is evidence of a direct contribution of morphological awareness, both in children (e.g., Gottardo et al., 2018) and adults (e.g., Guo et al., 2011), suggesting that the effect of this skill on reading comprehension is still important in adulthood. Accordingly, we hypothesized that the effect of morphological awareness will not be completely mediated by word reading and reading fluency, showing a direct path to reading comprehension.

In short, the present study aims to investigate an extended SVR model for reading comprehension in European Portuguese adult typical readers. It is relevant to recognize which abilities reading comprehension relies on to contribute to the identification of worthy targets of intervention to promote reading comprehension.

## Materials and Methods

### Participants

Sixty-seven adults (54 females, 80.6%), with ages ranging from 19 to 47years old (*mean*±*standard deviation*: 21.9±4.4) participated in this study. All participants had European Portuguese as their first language. Formal schooling ranged from 12 to 23years (*mean*±*SD*: 14.4±1.7). Most participants were college students (86.6%).

Exclusion criteria for participants were (1) previous diagnosis of reading, neurological, psychiatric or psychologic disorder and (2) scoring above 60 in the Adult Reading History Questionnaire (Lefly and Pennington, 2000; Portuguese version: *Questionário de Hábitos de Leitura*, Alves and Castro, 2005), a self-reported measure of reading difficulties.

### Measures

#### Reading Comprehension

A reading passage (Stocker, 2016) was translated into Portuguese and further adapted. The text had 495 words and was titled “Anne Frank.” Reading comprehension questions were developed according to the taxonomy of Day and Park (2005) and scoring criteria were agreed upon between the authors.

Three domains of reading comprehension were assessed: literal, inferential, and vocabulary. Literal comprehension questions were about facts in the text (eight questions). Inferential comprehension questions were divided into those where the participant had to infer based on implicit textual information (intratextual inference; four questions) and those where the participant had to activate background knowledge (extratextual inference; four questions). Vocabulary questions assessed the ability to deduce the meaning of an ambiguous word in context (four questions). Each one of four vocabulary words had two or more possible meanings, and only one was considered correct for the respective context.

Participants had to silently read the text and then answer aloud to comprehension questions. Silent reading was chosen because it is expected to foster comprehension, as the reader can allocate most cognitive resources to extracting meaning, instead of pronunciation or prosody (Hale et al., 2011). Participants could refer back to the text at any time during questioning and questions could be repeated if the participant did not understand them. The order of the questions was fixed for all participants and there was no time limit to answer.

Answers were scored with 0, 1, or 2 points, if the answer was completely incorrect, partially correct, or completely correct, respectively. Reading comprehension was computed as the sum of the obtained points, with a possible maximum score of 40. Cronbach’s alpha for this measure was 0.49, showing poor reliability. However, this reading comprehension score showed a significant positive correlation with the 1-min TIL (*r*=0.47; *p*<0.001), a validated measure of reading comprehension in adults (Fernandes et al., 2017).

#### Oral Language Comprehension

In studies comparing oral language and reading comprehension, measures should be well-calibrated with one another (Braze et al., 2007). Thus, an effort was made to equate these tasks, regarding the assessed domains (literal, inferential, and vocabulary) as well as the scoring procedure. For this task, six passages about the Portuguese poet Fernando Pessoa’s biography were adapted from Vilas-Boas and Vieira (2017). All passages had a similar length (*mean* number of words±SD=42.17±8.4, *range*=35–55). Twelve comprehension questions were created, two for each passage. However, questions 1 (passage 1) and 5 (passage 3) were later removed from the analysis due to clear ceiling effects. The questions assessed literal comprehension (two questions), knowledge of vocabulary in context (three questions), intrapassage inference (inference based on the information present on the passage; three questions), and extrapassage inference (inference based on previous knowledge; two questions). The selected vocabulary words had two or more possible meanings, and only one was considered correct. The frequency of these vocabulary words was similar for Oral Language and Reading Comprehension tasks (*mean*=17.5 and 21.8 occurrences per million, respectively, according to the P-PAL lexical database; Soares et al., 2018).

The passages were recorded by a male voice and played twice through headphones. The passages were repeated to reduce working memory constraints. The instructions and auditory stimuli were presented using the Presentation® software (version 21.1). A sheet with the comprehension questions was provided to the participants, at the beginning of the task. It was explained that they had to respond orally to those questions, based on the information present on auditory passages. The participants could silently read the questions beforehand and during the listening of the passages to scan them for relevant information. After answering the questions for a specific passage, participants pressed the space bar to listen to the next passage.

Answers were scored with 0, 1, or 2 points. The sum of the obtained points (maximum of 20) was taken as an oral language comprehension measure. This composite score showed poor reliability (Cronbach’s alpha=0.40).

#### Word Reading

The Reading Fluency Subtest of ADLER Battery (Faísca et al., 2019) was used to assess word reading abilities. This subtest includes five lists (high-frequency words, low-frequency words, consistent words, inconsistent words, and pseudowords) that the participants should correctly read as fast as possible during 30s. Word reading is an accuracy measure computed as the percentage of correctly read words on the four real word lists; this composite measure showed good internal consistency (Cronbach’s alpha=0.61; Faísca et al., 2019).

#### Phonological Decoding

Phonological Decoding is an accuracy measure computed as the percentage of correctly read pseudowords on the pseudoword list from the ADLER’s Reading Fluency Subtest. Test–retest correlation suggests weak reliability (*r*=0.24; Faísca et al., 2019).

#### Reading Fluency

Reading fluency is a speed measure computed as the average number of correctly read items across the five lists from the ADLER’s Reading Fluency Subtest. This composite measure has excellent internal consistency (Cronbach’s alpha=0.92) and good temporal stability (test–retest correlation: *r*=0.67; Faísca et al., 2019).

#### Phonological Awareness

Three phonological awareness tasks were used (phoneme deletion, spoonerisms, and phonological acronyms; Faísca et al., 2019). All tasks have good reliability (Cronbach’s alphas ranging from 0.70 to 0.90) and showed moderate to strong correlations (mean *r*=0.53; all *p*<0.01), so a composite measure for phonological awareness was computed based on the average of the z-transformed accuracy scores from each task. This composite measure has excellent temporal stability (test–retest correlation: *r*=0.85; Faísca et al., 2019).

#### Rapid Automatized Naming

Digit and letter naming tasks were used (Alves et al., 2007) since RAN alphanumeric measures have been considered as stronger predictors of reading-related skills than non-alphanumeric measures (e.g., Araújo et al., 2015; Donker et al., 2016). As these tasks correlated strongly (*r*=0.74), a RAN composite was computed, representing the average number of correctly named items per second. The Spearman-Brown coefficient was *r*=0.84, indicating good reliability for this measure.

#### Morphological Awareness

Two morphological awareness computer-driven tasks were developed based on Cavalli et al. (2017): the Suffixation Decision Task and the Suffixed Word Detection Task. These tasks were designed to assess explicit morphological awareness since they required extracting the stem word from a derived form (Martin et al., 2014).

In these tasks, all words were nouns, in the singular form, and had a regular grapheme-phoneme conversion, to ensure that performance was based exclusively on morphology. Suffixation stimuli were matched for phonological/orthographic shift (Wilson-Fowler and Apel, 2015), as well as for word length (3–4 syllables).

All the words were audio-recorded and played through headphones to prevent the participants to extract the stem word through orthographic analysis of the stimulus, and thus avoiding possible cofounding with word reading skills (Cavalli et al., 2017). Before performing the morphological awareness tasks, all participants were instructed on the definitions of stem words, affixes (suffixes and prefixes), suffixed and prefixed words, and pseudosuffixed and pseudoprefixed words. Morphological awareness tasks were always presented in the same order.

##### Suffixation Decision Task

Thirty-two words were used as auditory stimuli, half being morphologically complex and suffixed (e.g., “carteiro”/postman) and half being morphologically simple and pseudosuffixed (e.g., “dinheiro”/money). Pseudosuffixed words have a suffix-like ending (e.g., “-eiro”) but are monomorphemic. Frequency and word length were matched between suffixed and pseudosuffixed items. Immediately after the auditory presentation of each stimulus, participants should decide as fast and accurately as possible if the word was suffixed or not. The item presentation order was pseudorandomized and fixed across participants. Before the task, participants were trained with four examples, and oral feedback was given. Accuracy scores were calculated as the percentage of correctly answered items.

##### Suffixed Word Detection Task

Words were organized in 12 triplets (groups of three words), comprising one suffixed word and two pseudosuffixed words (e.g., “ossada, geada, cilada”/bone, frost, trap, being “ossada” the suffixed target). Frequency and word length were matched between suffixed and pseudosuffixed items. Words within triplets were auditorially presented one by one, with a one-second pause between words. Triplets were always presented twice, with 2seconds between them, to avoid working memory constraints. Immediately after hearing the triplet for the second time, participants had to detect the word that was suffixed, by pressing either the 1, 2, or 3 button keys on the computer keyboard, if the target suffixed word was the first, second, or third item of the triplet. Participants were instructed to respond as fast and accurately as possible with their preferred hand. The presentation order of the triplets was pseudorandomized and fixed across participants. Before the task, participants trained with two example triplets, and oral feedback was given. Accuracy scores were calculated as the percentage of correctly answered items.

The morphological awareness score was computed averaging the z-transformed accuracy scores obtained in the Suffixation Decision and the Suffixed Word Detection Tasks.

#### Auditory Working Memory

The backward condition of the Digit Span subtest of the WAIS-III (Portuguese version; Wechsler, 2008) was used to assess working memory, considering that this task requires storage and manipulation of auditory information (Novaes et al., 2019). The raw scores were used as a working memory measure.

#### Vocabulary

The Vocabulary subtest of the WAIS-III (Portuguese version; Wechsler, 2008) was used to measure oral vocabulary knowledge. In the present work, raw scores were converted to standardized scores, based on the WAIS-III age groups and used as a vocabulary knowledge measure.

### Procedure

This study is part of a larger research project aiming at the development and validation of a battery of tests to assess reading and reading-related skills in European Portuguese adults (the ADLER Battery; Faísca et al., 2018, 2019). The participants were recruited among those who were being assessed in the ADLER sessions. Typical adult readers were selected and asked to collaborate in the present study. For those who agreed, an additional session took place, to administer the new tasks not included in the ADLER Battery (reading comprehension, oral language comprehension, and morphological awareness). The order of administration of the tasks was fixed for all participants.

Before the administration of the tasks, participants gave their informed consent, according to the current Portuguese personal data protection law. Participants also filled a questionnaire with relevant sociodemographic information.

### Data Analysis

Regression and path analyses approaches were used to test SVR and mediation models. Path analysis is a statistical method developed to study simultaneously the direct and indirect effects of a set of independent variables on one or more dependent variables (Streiner, 2005), providing estimates of the magnitude of the hypothesized relationships (*paths*) among variables.

Path coefficients point estimates (unstandardized and standardized) were complemented with bootstrap percentile confidence intervals, BPCI (based on 2,000 samples). To assess the goodness-of-fit of the path models, we used the Chi-squared statistic (*X*^2^), the Comparative Fit Index (CFI), and the Root Mean Square Error of Approximation (RMSEA). CFI values higher than 0.9 indicate an acceptable fit, while RMSEA should be lower than 0.05 to verify a good fit, with values between 0.05 and 0.08 suggesting a reasonable fit (Hu and Bentler, 1999).

To test the mediation hypothesis, a full mediation model (direct effects were restricted to zero, except those involving the mediator) was estimated first, to check for non-null indirect effects. If indirect effects existed, the full mediation model was compared to the partial mediation model (where direct effects are freed). Significant goodness-of-fit differences between both models would indicate that restricting the direct effects to zero hinders the model’s adjustment, and so the total mediation model cannot be accepted, and direct paths should be maintained (partial mediation). Contrarily, non-significant differences between models would indicate that restricting the direct effects to zero does not hinder the model’s adjustment and so full mediation can be assumed. Chi-squared tests were used to assess the significance of the difference between the goodness-of-fit of nested models.

Besides the path analyses, descriptive and correlational statistics were performed. The guidelines of Cohen (1988) for the strength of correlations in behavioral sciences were followed. All data were processed using the IBM SPSS Statistics (v.26) and IBM SPSS AMOS (v.26) software.

## Results

### Descriptive Statistics

Table 1 shows the descriptive statistics for all variables in the study. According to Kline’s (2005) suggestion, the skewness and kurtosis coefficients indicate no severe deviation from normality in the variable distributions. Scores on morphological and phonological awareness measures were somewhat skewed to the left, but the visual inspection of their distribution (boxplot and histogram) indicates that the relatively high concentration of scores on the right may not be considered a ceiling effect.

**Table 1:** Correlation matrix (Pearson product–moment correlation coefficients) and descriptive statistics.

	1	2	3	4	5	6	7	8	9	10
1. RAN	1									
2. Morphological awareness	−0.01	1								
3. Phonological decoding	0.08	0.17	1							
4. Phonological awareness	0.09	0.35**	0.33**	1						
5. Working memory	0.06	0.05	0.19	0.50**	1					
6. Word reading	0.06	0.18	0.40**	0.29*	0.26*	1				
7. Reading fluency	0.47**	0.15	0.23	0.26*	0.34**	0.34**	1			
8. Vocabulary	0.14	0.15	0.02	0.35**	0.34**	0.36**	0.31*	1		
9. Oral lang. comprehension	0.07	0.23	0.00	0.23	0.37**	0.27*	0.25*	0.27**	1	
10. Reading comprehension	0.11	0.34**	0.06	0.35**	0.36**	0.34**	0.30*	0.42**	0.47**	1
Mean	3.04	0.00	93.77	0.00	7.13	96.83	1.67	10.67	11.47	24.34
standard deviation	0.46	0.83	5.15	0.83	2.12	1.55	0.26	2.56	2.35	4.23
Skewness	0.326	−1.028	−0.638	−2.034	0.105	−0.860	−0.033	0.010	0.164	0.144
Kurtosis	−0.592	2.455	−0.266	6.527	−0.066	2.271	−0.317	2.603	−0.218	−1.025

RAN – Number of correctly named items per second; Morphological Awareness – Accuracy (z-scores); Phonological Decoding – Percentage of correctly read pseudowords; Phonological Awareness – Accuracy (z-scores); Working Memory – Raw scores (max=14); Word Reading – Percentage of correctly read words; Reading Fluency – Number of correctly read words per second; Vocabulary - Standardized scores; Oral Language Comprehension – Number of correct answers (max=20); Reading Comprehension – Number of correct answers (max=40); *p<0.05 and **p<0.01.

Z-scores for measures of phonological decoding (*mean*=0.18, min=−2.58, max=1.36), word reading (*mean*=0.19, min=−3.20, max=2.06) and reading fluency (*mean*=0.13, min=−2.65, max=3.00) were computed based on the scores of 150 typical adult readers (Faísca et al., 2019) and they indicate that, on average, the present sample does not deviate from the expected performance level on these tasks.

Pearson correlations among predictors were always positive (except for the null correlation between RAN and morphological awareness, *r*=−0.01, *p*=0.918), but not always significant. Significant correlations among predictors ranged from weak to moderate (0.25<*r*<0.50). Predictors correlated significantly with reading comprehension, with the exceptions of phonological decoding and RAN. All significant correlations between predictors and reading comprehension were positive and moderate, ranging from 0.30 (reading fluency) to 0.47 (oral language comprehension).

### SVR Model

**Figure 3:**
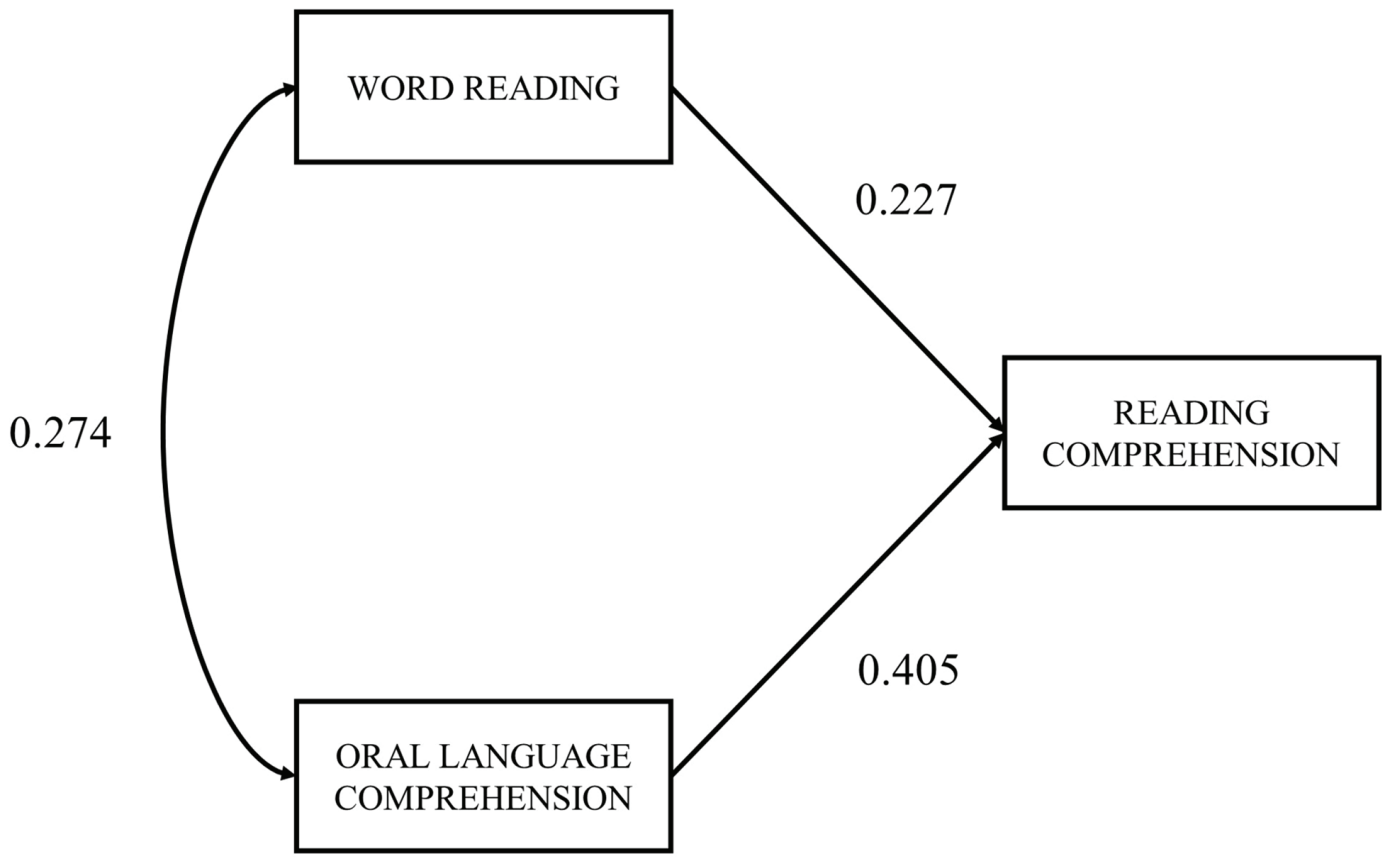
SVR model with standardized path coefficients. All paths are significant (*p*<0.05).

The SVR model (Figure 3) is a saturated model (degrees of freedom=0), so the goodness of fit indexes could not be computed. Both word reading (standardized coefficient *β*=0.227; 95% BPCI [0.038, 0.391]) and oral language comprehension (standardized coefficient *β*=0.405; 95% BPCI [0.157, 0.713]) have a significant direct effect on reading comprehension. Together, these two predictors explained about 27% of the variance in reading comprehension (*R*^2^=0.266). Although the standardized coefficient for oral language comprehension seems to express a somehow greater effect on reading comprehension compared to word reading, pairwise parameter comparison showed that this difference was non-significant (critical ratio=0.27, *p*>0.7). Confidence intervals for path coefficients were rather wide and overlapped, suggesting that the magnitude of these effects cannot be considered reliably different.

### Extended SVR Model

A hierarchical regression analysis with two blocks was used to test if reading fluency and vocabulary could add a significant contribution to the SVR model. The first block contained the two main components of the SVR (word reading and oral language comprehension), and the second block included the reading fluency and vocabulary measures. This regression model provided a significant addition of near 7% above the reading comprehension variance explained by the SVR model [*R*^2^=0.335; *R*^2^ change=0.069; *F* change (2, 62)=3.2, *p*=0.046]. In this extended model, the effect of vocabulary on reading comprehension was significant (*β*=0.256, *p*=0.030) but the effect of reading fluency was not (*β*=0.091, *p*=0.429). Also, the effect of word reading on reading comprehension was attenuated, losing its significance when reading fluency and vocabulary were considered (*β*=0.227, *p*=0.045 in the first block and *β*=0.122, *p*=0.297 after including the second block). The effect of oral language comprehension on reading comprehension maintains its significance even in the presence of reading fluency and vocabulary (*β*=0.342, *p*=0.003).

**Figure 4:**
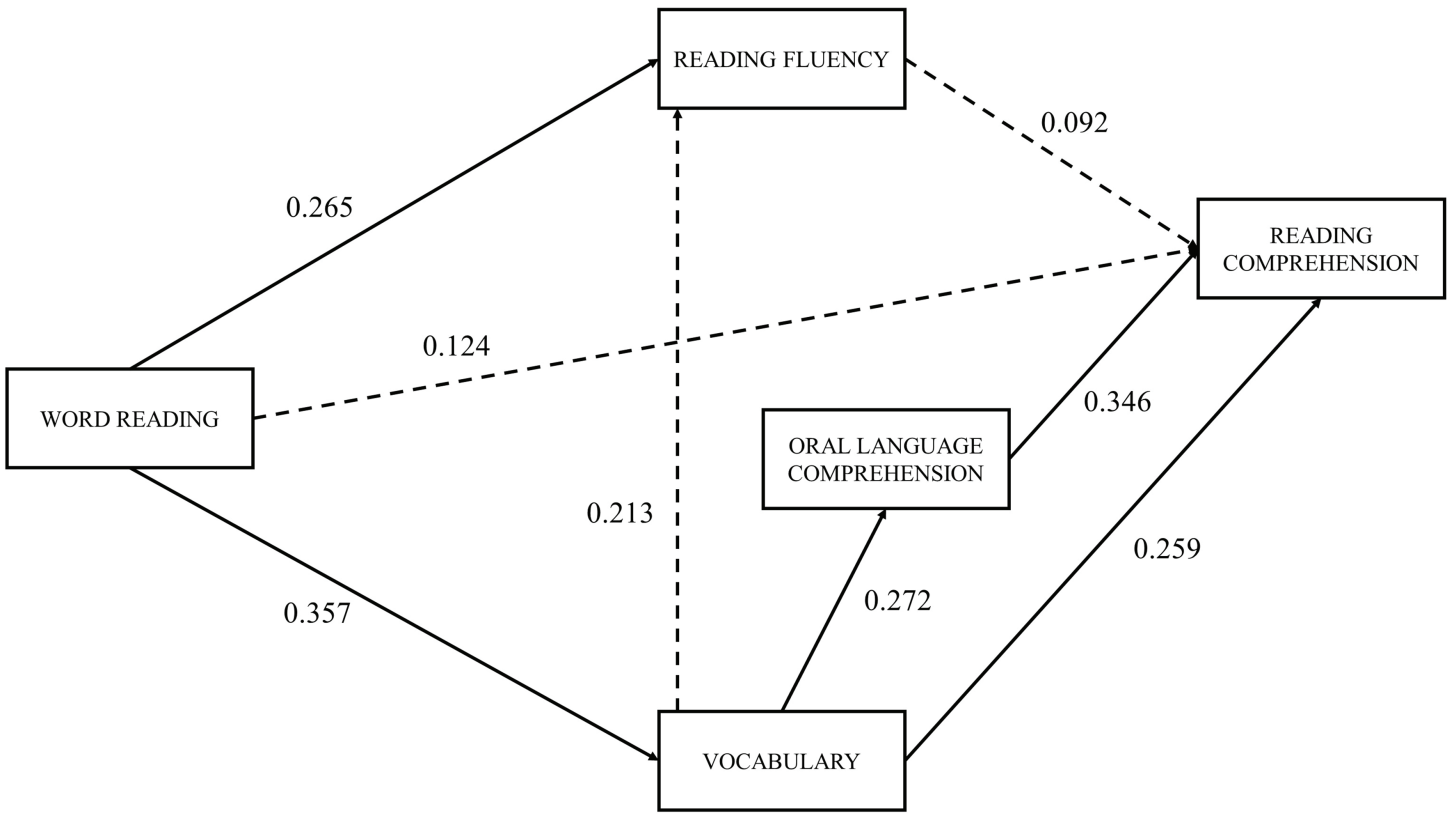
Extended SVR model with standardized path coefficients. *χ*^2^ (2)=3.814, *p*=0.149; CFI=0.961; RMSEA=0.117. Dashed lines represent non-significant paths; solid lines represent significant paths (*p*<0.05).

The path analysis of the extended SVR model (Figure 4) helps to elucidate the consequences of including reading fluency and vocabulary as predictors of reading comprehension. While chi-square goodness-of-fit statistic [*X*^2^ (2)=3.8, *p*=0.149] and the CFI=0.961 suggests a good model fit, the RMSEA=0.117 indicates poor adjustment. However, considering that RMSEA is known to be too restrictive when the model has a small number of degrees of freedom and the sample size is small (Kenny et al., 2015), and considering the Chi-square and CFI indexes, we can assume that the extended SVR model depicted in Figure 4 represents the sample data adequately.

Five out of the eight hypothesized paths of the extended SVR model were significant (Table 2). Lastly, Table 3 shows the standardized direct, indirect, and total effects of the variables on reading comprehension. Overall, word reading does not have a direct effect on reading comprehension, exerting its indirect influence mainly through vocabulary. Vocabulary influences reading comprehension both directly and through oral language comprehension.

**Table 2:** Unstandardized and standardized path coefficients for the extended SVR model.

Paths	Unstandardized	Standard error	Standardized	*p*
Word reading → Reading fluency	0.045	0.020	0.265	0.028
Word reading → Vocabulary	0.592	0.190	0.357	0.002
Vocabulary → Reading fluency	0.022	0.012	0.213	0.078
Vocabulary → Oral language comprehension	0.250	0.109	0.272	0.021
Word reading → Reading comprehension	0.079	0.072	0.124	0.272
Reading fluency → Reading comprehension	0.349	0.421	0.092	0.407
Vocabulary → Reading comprehension	0.100	0.044	0.259	0.024
Oral lang. comprehension → Reading comprehension	0.145	0.044	0.346	0.001

**Table 3:** Standardized direct, indirect, and total effects of predictors on reading comprehension, in the extended SVR model.

Predictors	Direct (*p*)	Indirect (*p*)	Total (*p*)
Word reading	0.124 (0.169)	0.158 (0.019)	0.281 (0.017)
Reading fluency	0.092 (0.438)	-	0.092 (0.438)
Vocabulary	0.259 (0.018)	0.114 (0.035)	0.373 (0.010)
Oral language comprehension	0.346 (0.019)	-	0.346 (0.019)

### Effects of the Remaining Predictors on Reading Comprehension

**Figure 5:**
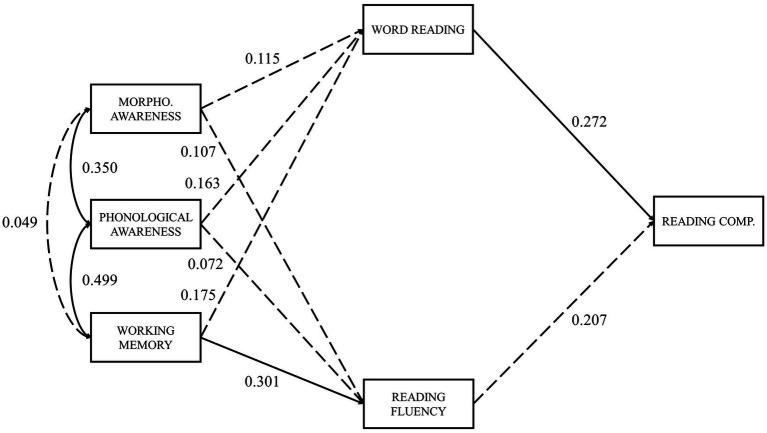
Full mediation by word reading and reading fluency model, with Standardized path coefficients. *χ*^2^ (4)=15.641, *p*=0.004; CFI=0.800; RMSEA=0.210. Dashed lines represent non-significant paths; solid lines represent significant paths (*p*<0.05).

**Figure 6:**
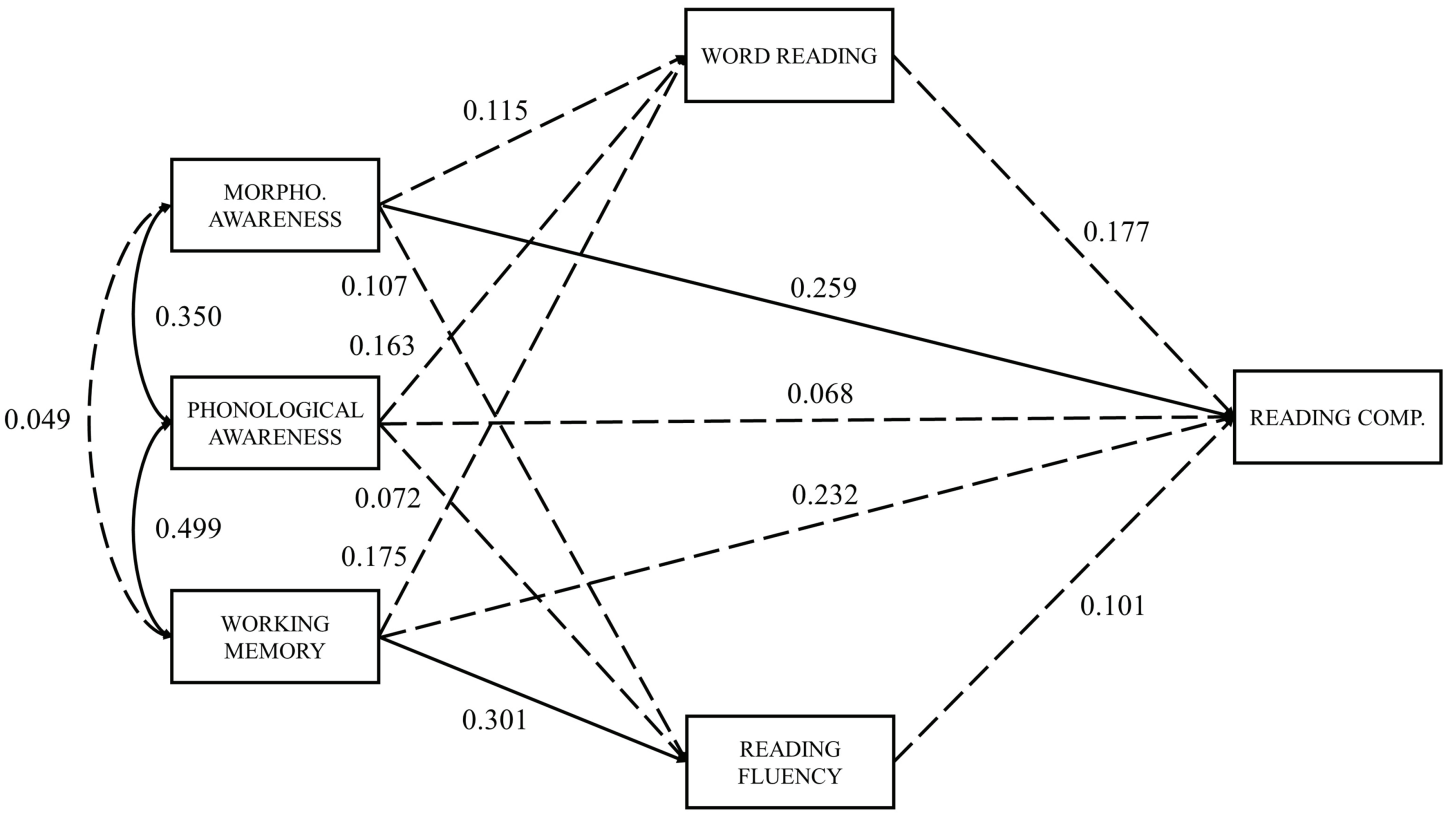
Partial mediation by word reading and reading fluency model with standardized path coefficients. *χ*^2^ (1)=4.417, *p*=0.036; CFI=0.941; RMSEA)=0.228. Dashed lines represent non-significant paths; solid lines represent significant paths (*p*<0.05).

Since RAN and phonological decoding did not correlate significantly with reading comprehension, both measures were not included in the mediation analyses. Thus, the effects of phonological awareness, morphological awareness, and working memory on reading comprehension were tested, to verify if direct effects on reading comprehension do exist, or if these effects were totally mediated by word reading and reading fluency. To test our mediation hypotheses, two models were tested: full mediation through word reading and reading fluency (model 1a, Figure 5) and partial mediation through word reading and reading fluency (model 1b, Figure 6).

Chi-square statistics were significant for both models (*p*<0.05), indicating a poor fit (Table 4). CFI indicated a good fit only for the partial mediation model (CFI>0.9). Again, as expected due to the small number of degrees of freedom (Kenny et al., 2015), the RMSEA index suggests a poor fit (RMSEA>0.2) for both models. However, the crucial step in this analysis is to compare the full and partial mediation models. The difference in chi-square statistics between the two models was significant (*p*=0.011), suggesting that word reading and reading fluency did not completely mediate the effects that morphological awareness, phonological awareness, and working memory may have on reading comprehension.

**Table 4:** Goodness-of-fit indexes for the mediation models and comparison between full and partial mediation models.

Models	χ^2^(df), *p*	CFI	RMSEA	Comparisons
1a – Full mediation by word reading and reading fluency	15.6 (4), 0.004	0.800	0.210	-
1b – Partial mediation by word reading and reading fluency	4.4 (1), 0.036	0.941	0.228	Δχ^2^ =11.224, Δdf=3, *p* =0.011

*df=degrees of freedom; CFI=Comparative Fit Index; RMSEA=Root Mean Square Error of Approximation*.

In the full mediation model, all indirect effects on reading comprehension through word reading and reading fluency were non-significant, except for working memory (model 1a; *β*=0.110, *p*=0.045). When direct effects were allowed (model 1b), only morphological awareness revealed a significant direct impact on reading comprehension (model 1b; *β*=0.259, *p*=0.023). Phonological awareness showed no significant direct or indirect effects on reading comprehension (Table 5).

**Table 5:** Standardized direct, indirect, and total effects of predictors on reading comprehension, in the full mediation (1a) and partial mediation (1b) models.

Predictors	Model 1a			Model 1b		
	Direct (*p*)	Indirect (*p*)	Total (*p*)	Direct (*p*)	Indirect (*p*)	Total (*p*)
PA	-	0.059 (0.273)	0.059 (0.273)	0.068 (0.575)	0.036 (0.259)	0.104 (0.436)
MA	-	0.053 (0.282)	0.053 (0.282)	0.259 (0.023)	0.031 (0.280)	0.291 (0.025)
WM	-	0.110 (0.045)	0.110 (0.045)	0.232 (0.114)	0.062 (0.109)	0.293 (0.032)
WR	0.272 (0.035)	-	0.272 (0.035)	0.177 (0.167)	-	0.177 (0.167)
RF	0.207 (0.073)	-	0.207 (0.073)	0.101 (0.382)	-	0.101 (0.382)

PA=Phonological Awareness; MA=Morphological Awareness; WM=Working Memory; WR=Word Reading; RF=Reading Fluency. Model 1a – full mediation through word reading and reading fluency; Model 1b – partial mediation through word reading and reading fluency.

## Discussion

Research on the predictors of reading comprehension has been largely focused on school-aged children, and in more opaque orthographies, such as English. These studies cannot be fully generalized to adults typical readers, that rely on different cognitive processes when reading (Greenberg et al., 2002), or to more transparent orthographies, as transparency affects the weight of the contribution of predictors on reading comprehension (Florit and Cain, 2011). Moreover, the SVR model, despite some cases of high percentages of explained variance of reading comprehension in both children (e.g., Catts et al., 2005) and adults (e.g., Sabatini et al., 2010), has been often criticized for being too simplistic, and other components have been suggested, such as vocabulary and reading fluency.

In this study, we set out to examine the relations between several reading-related predictors and reading comprehension in European Portuguese-speaking adults. For that, we selected a set of predictors identified in the meta-analysis of Tighe and Schatschneider (2016), namely oral language comprehension and word reading (as it is assumed in SVR), vocabulary and reading fluency (frequently included in SVR extended models), and phonological decoding, phonological awareness, rapid automatized naming, working memory and morphological awareness. As expected, our results showed that all these predictors correlated significantly, positively, and moderately with reading comprehension, with the exceptions of phonological decoding and RAN. The absence of a significant correlation between phonological decoding and reading comprehension could be partially explained by the relative transparency of the European Portuguese orthography in the print-to-read conversion. In semitransparent orthographies such as Portuguese, the grapheme-phoneme conversion is simpler, allowing readers to achieve fluent decoding in the first school years (Seymour et al., 2003). When fluent reading is achieved, reading performance no longer depends on grapheme-phoneme conversions, and therefore correlations between phonological decoding and reading comprehension lose strength. This might explain the null correlation between phonological decoding and reading comprehension in the present study.

The absence of correlation between RAN and reading comprehension could result from reading expertise. Tighe and Schatschneider (2016) contrasted the correlations in their meta-analysis with correlations reported in a large meta-analytical study addressing the predictors of reading comprehension in early childhood and kindergarten (National Early Literacy Panel, 2008). The authors found that RAN was weakly related to reading comprehension in their reviewed studies with adult readers (average *r*=0.15), but this correlation had a moderate magnitude in the studies reviewed by the National Early Literacy Panel (2008) (average *r*=0.43). These findings suggest that the association between RAN and reading comprehension loses its strength in adulthood and that this association depends on reading expertise. Since RAN is a well-known predictor of reading fluency (Savage and Frederickson, 2005), it will affect reading comprehension probably in an indirect manner, *via* reading fluency. In early school years, while fluent reading is not yet achieved, reading fluency and RAN prove to be important predictors of reading comprehension. However, in higher grades, readers have already achieved proficient reading, and consequently reading fluency will show a reduced effect on comprehension. If this is the case, it might explain why the effect of RAN on reading comprehension is absent in our adult sample.

Regarding the simple SVR model, our results demonstrated that both word reading and oral language comprehension displayed direct and significant effects on reading comprehension, with the latter showing a stronger effect, and apparently confirming our hypothesis. However, inferential procedures indicate that this difference cannot be considered as statistically reliable (perhaps due to the lack of statistical power). Despite that, there is a clear tendency that arose from previous studies (e.g., Florit and Cain, 2011; Cadime et al., 2017) that allow us to suggest that in a sample with advanced grade levels and for an orthography of intermediate transparency, oral language comprehension should provide a significantly higher contribution than word reading to reading comprehension.

The two components of the SVR model only explained about 27% of the variance in reading comprehension, contrasting with the values found in the literature, usually higher (e.g., 74% in Braze et al., 2007; 64% in Sabatini et al., 2010). A possible explanation for such differences might result from the samples used in previous studies, namely English adult struggling readers, whose reading comprehension might still be strongly dependent on word decoding processes. Therefore, the comparison with such populations of struggling readers should be done with precaution. In typical English adult readers, the SVR model explains a fraction of the reading comprehension variance similar to the observed in our sample (34% of the explained variance; Macaruso and Shankweiler, 2010). Another possible explanation is the exclusive reliance on observable variables (e.g., Sabatini et al., 2010; Braze et al., 2016), an approach that diminishes measurement error and allows more reliable measures. Furthermore, reliability coefficients for our oral language and reading comprehension tasks were low (Cronbach’s alpha=0.40 and 0.49, respectively), so in the future, we should consider adopting methods to improve the reliability of our measures, to lessen measurement errors and hence proving more accountability for the variance in reading comprehension.

The extended SVR model included reading fluency and vocabulary and provided a significant addition to the explained variance in reading comprehension (7%). Nonetheless, vocabulary was the only one of the two added variables that showed a significant individual contribution to reading comprehension. The inclusion of these new variables also caused the direct effect of word reading to become non-significant, demonstrating that word reading only affects reading comprehension indirectly. A more detailed analysis showed that this indirect effect happens mostly *via* vocabulary. Thus, at least in our adult sample, word reading accuracy effects on reading comprehension might reflect the reciprocal association between reading accuracy and the acquisition of new word meanings (Ricketts et al., 2007; Mellard et al., 2010).

The direct effect of vocabulary on reading comprehension was expected. A study performed with Portuguese children suggests that while reading fluency remains important from the first to the sixth grade, vocabulary emerges as a significant predictor in the second grade, gaining importance throughout the school years, as reading fluency loses relevance (Fernandes et al., 2017). By the sixth grade, vocabulary’s importance catches up with reading fluency’s, and this tendency could go on as the reader advances in schooling, with reading becoming more fluent and vocabulary size increasing. Indeed, in our sample, reading fluency was not a significant predictor of reading comprehension, while vocabulary showed significant direct and indirect effects (through oral language comprehension). Once again, this suggests that, at least in more transparent orthographies, decoding skills are important in early school years, until reading becomes fluent. Then, higher-order skills such as vocabulary emerge and remain important to achieve reading comprehension, throughout schooling and adulthood.

The effect of vocabulary on reading comprehension, in our study, provides support for its addition as a separate component in the SVR model. Other studies that used path analysis (e.g., Mellard et al., 2010) or regression models (e.g., Braze et al., 2007) also support this idea. However, Braze et al. (2016) proposed that the observed effect of vocabulary on reading comprehension could be explained by the typical low-reliability of oral language comprehension measures, which might not be capturing all aspects that are relevant for reading comprehension, which in turn might be apprehended by the more reliable vocabulary measures. Since our oral language comprehension measure presented low reliability, this may be also the case for our study. Thus, this significant effect of vocabulary on reading comprehension should be interpreted with caution, until other studies, with different statistical procedures or more reliable measures of oral language comprehension, can confirm vocabulary relevance in the SVR model for the studied population.

Although the results of the present study show that the SVR model (with the possible addition of vocabulary) can reliably predict reading comprehension in adults, the percentage of explained variance by the model is smaller than the reported in previous studies with English struggling adult readers. This difference might be due to both the different levels of reading expertise of the studied samples or to the orthographies’ transparency. More studies are needed to verify the SVR’s adequacy in adult typical readers, and they should include proposals for additional inclusions as a way of increasing the percentage of explained variance of reading comprehension. Recent studies have suggested the inclusion of higher-order cognitive skills (e.g., inference making, perspective-taking, and comprehension monitoring; Kim, 2017, 2020), text characteristics (e.g., sentence length and frequency of the words; Francis et al., 2018), and variables of self-regulation when reading (e.g., motivation, engagement, and the use of reading strategies; Duke and Cartwright, 2021).

In the final mediation analyses, we tested if the effects of the remaining variables at study (morphological awareness, phonological awareness, and working memory) on reading comprehension were direct or mediated by word reading and reading fluency. The total mediation hypothesis was rejected, suggesting that word reading and reading fluency did not completely mediate the contribution of these predictors. As expected, morphological awareness was the only variable that presented a significant direct effect on reading comprehension. In the meta-analysis of Tighe and Schatschneider (2016), morphological awareness was the strongest predictor of reading comprehension. According to Kirby et al. (2008), this skill gains importance as the reader progresses to more advanced levels of schooling. As text exposure increases, so does the number of morphologically complex words that the reader is exposed to, providing more opportunities for the use of morphological awareness skills. The direct effect of morphological awareness on reading comprehension, in adults, can be observed in more opaque orthographies such as English (see, for example, Wilson-Fowler and Apel, 2015; Fracasso et al., 2016). In such orthographies, since grapheme-phoneme conversion is not consistent, the ability to manipulate morphemes aids in accurately reading morphologically complex words and comprehending texts. In more transparent orthographies, such as Portuguese, decoding is easier, since grapheme-phoneme conversion is more consistent, and therefore morphological awareness is not so relevant to accurate reading, but still plays an important role in meaning-extraction to achieve comprehension of what was read.

Working memory showed a significant total effect on reading comprehension, in the final mediation models, although individual direct and indirect paths were non-significant. This is an indicator that individual effects might be important, as their sum reaches statistical significance. Indeed, a direct effect of working memory on reading comprehension would be expectable, since working memory allows readers to store and manipulate information from the text as they read and integrate it with previously stored knowledge (Daneman and Merikle, 1996). In addition, an indirect effect of working memory on reading comprehension, through word reading and reading fluency, makes theoretical sense. The larger the amount of information that readers can store and process continuously, the more accurate and faster they can read since they can quickly retrieve word pronunciations and meanings from their long-term memory.

Surprisingly, phonological awareness did not show a significant direct or indirect path of influence to reading comprehension in the final mediation models, even though it correlated significantly with reading comprehension. An explanation we could provide for this is that phonological awareness and working memory correlated moderately (*r*=0.50, *p*<0.01), sharing explained variance. This correlation probably reflects the working memory demands of phonological awareness tasks, where participants typically need to store and manipulate verbal information of increasing difficulty. In this way, phonological awareness could be reflecting the effects of working memory on reading comprehension, lessening its effect when the two predictors are considered together. In the future, other studies should try to disentangle the relations between these variables and reading comprehension.

This study was the first one to investigate the predictors of reading comprehension in a sample of European Portuguese-speaking adults, and so several measures were specifically tailored for this study. Consequently, our findings should be interpreted taking into account the low reliability of some tasks. Also, our relatively small sample size only provided statistical power to detect moderate effects on reading comprehension. A larger sample should contribute with sufficient statistical power to detect smaller but still relevant effects. Additionally, considering the predominance of female participants in our study, our results should be confirmed in a more gender-balanced sample.

We consider that the greatest implication of the present work is that it provides a re-thinking about the models of reading comprehension for typical adult readers, in a less opaque orthography such as European Portuguese. Future investigations might use these results as a term of comparison with other age-groups, education levels, reading skills, and orthographies, or as a way of identifying relevant targets of intervention for the improvement of reading comprehension levels in Portuguese adults. Oral language comprehension and vocabulary were the best predictors of reading comprehension in the present study and therefore these abilities can be the target of remediation programs to increase reading comprehension levels, in adults.

In sum, this study adds evidence that the transparency of the orthography and reading expertise affect the relative contribution of predictors on reading comprehension. Results show that the SVR model (with the significant addition of vocabulary) could be an adequate model to predict reading comprehension in typical adult readers in a semitransparent orthography, even though other variables could probably increase the percentage of explained variance in reading comprehension.

## Data Availability Statement

The raw data supporting the conclusions of this article will be made available by the authors, without undue reservation.

## Ethics Statement

Ethical review and approval was not required for the study on human participants in accordance with the local legislation and institutional requirements. The patients/participants provided their written informed consent to participate in this study.

## Author Contributions

FG, AR, and LF: conceptualization and methodology. FG, IS, and LF: investigation and formal analysis. FG, AR, FI, and LF: writing – original draft. AR, FI, and LF: writing – review and editing. AR: funding acquisition. All authors contributed to the article and approved the submitted version.

## Funding

This work was supported by the Portuguese Foundation for Science and Technology (FCT): PTDC/MHC-PCN/1175/2014 and UIDB/04255/2020 CINTESIS.

## Conflict of Interest

The authors declare that the research was conducted in the absence of any commercial or financial relationships that could be construed as a potential conflict of interest.

## Publisher’s Note

All claims expressed in this article are solely those of the authors and do not necessarily represent those of their affiliated organizations, or those of the publisher, the editors and the reviewers. Any product that may be evaluated in this article, or claim that may be made by its manufacturer, is not guaranteed or endorsed by the publisher.
